# Prevalence, patterns, and associated factors of work-related musculoskeletal disorders among intensive care unit and emergency room nurses in Palestine: a multicenter cross-sectional study

**DOI:** 10.3389/fpubh.2026.1865126

**Published:** 2026-07-14

**Authors:** Sana Al-Aqqad, Shatha Bani Odeh, Hadeel Qasqas, Yasmeen Alqaisi, Ali Aldirawi, Abdallah Alwawi

**Affiliations:** 1 Division of Nursing and Midwifery, Ibn Sina College for Health Professions, Nablus University for Vocational and Technical Education (NUVTE), Nablus, Palestine; 2 Center of Postgraduate Studies, School of Nursing, Applied Science, Lincoln University College, Selangor, Malaysia; 3 Department of Nursing, Faculty of Health Professions, Al-Quds University, Jerusalem, Palestine; 4 INTI International University, Nilai, Negeri Sembilan, Malaysia

**Keywords:** associated factors, emergency departments, intensive care units, nurses, occupational health, prevalence, work-related musculoskeletal disorders

## Abstract

**Background:**

Work-related musculoskeletal disorders (WMSDs) represent a leading occupational health concern among nurses globally. These disorders affect muscles, ligaments, bones, joints, and tendons, often leading to absenteeism, work restrictions, and reduced work efficiency. This study aimed to assess the prevalence of WMSDs among Palestinian nurses working in intensive care units (ICUs) and emergency rooms (ERs), identify the patterns of WMSDs, and examine the associated factors.

**Methods:**

A multicenter cross-sectional study was conducted in four main public hospitals in Palestine from 1 to 30 April 2024. A total population (census) sampling strategy was employed to ensure comprehensive coverage of the target population. The total eligible population consisted of 183 nurses, including 99 nurses working in ICUs and 84 nurses working in ERs. A self-administered questionnaire based on the Nordic Musculoskeletal Questionnaire (NMQ) was used to assess WMSDs. Data were analyzed using SPSS version 27, and the chi-square tests were used to examine unadjusted associations between WMSD symptoms and sociodemographic and work-related variables.

**Results:**

A total of 160 nurses completed the questionnaire, including 89 ICU nurses and 71 ER nurses, yielding an overall response rate of 87.4%. The overall prevalence of WMSDs in the past 12 months was 95.0%, 152 of 160. The within-department prevalence was 96.6% among ICU nurses, 86 of 89, and 93.0% among ER nurses, 66 of 71. The overall difference between departments was not statistically significant, chi-square = 1.121, *p* = 0.468. The most commonly affected anatomical sites were the lower back (85.0%), followed by the shoulders (77.0%), and the neck (71.3%). ER nurses reported a significantly higher prevalence of knee problems (51.6% vs. 48.4%, *p* = 0.035) and ankle/foot issues (54.1% vs. 45.9%, *p* = 0.017) compared to ICU nurses. Nurses who do not exercise regularly reported more frequent WMSD symptoms than those who exercise (*p* = 0.017). Additionally, nurses with 5–9 years of work experience reported higher WMSD rates compared to those with 1–4 years and more than 9 years (*p* = 0.029).

**Conclusion:**

Work-related musculoskeletal disorders were highly prevalent among Palestinian ICU and ER nurses, primarily affecting the lower back, shoulders, and neck. Years of work experience and lack of regular exercise were significantly associated with WMSDs, with notable differences between ICU and ER staff in knee and ankle/foot problems.

## Introduction

Work-related musculoskeletal disorders (WMSDs) are impairments of the musculoskeletal system, including muscles, joints, tendons, ligaments, nerves, cartilage, and bones, primarily caused by performing work tasks and the environment in which work is carried out (1). WMSDs are very common among healthcare professionals, particularly nurses, due to the physically demanding nature of nursing work and the high-risk environments (2). The annual prevalence of WMSDs among nurses globally was estimated to range from 77.2 and 81% (3, 4), with higher rates in developing countries compared to developed ones (4). Among Asian nurses specifically, the overall prevalence was reported at 84.3%, with the lower back being the most commonly affected region (5). It was estimated that between 43 and 78% of healthcare workers in the Arab world suffer from WMSDs (6). However, the prevalence was higher among nurses, ranging from 62.3 to 91%, such as in Egypt (7–10), Jordan (11), and Saudi Arabia (12), revealing a rate of 88, 91, and 63.8%, respectively. In Palestine, existing studies have either examined low back pain specifically among nurses in Nablus city (13) or investigated WMSDs among nurses working in conflict-affected hospitals in Gaza, reporting high prevalence, particularly in the neck, lower back, and shoulders (14). However, research on the full spectrum of WMSDs among nurses in the West Bank remains scarce, and no study has specifically targeted ICU and ER nurses across multiple centers, representing a significant gap that the present study aims to address.

The WMSDs experienced by nurses vary across wards, hospitals, and countries of employment (15). For example, compared to Oceanians, African and Asian nurses have three times the prevalence of WMSDs (16), while the risk is higher for ER nurses than for those working in ICUs, regular wards (15), supply rooms, surgery departments, and anesthesia sections (17). Previous studies revealed that more than 90% of nurses who work in ICUs experience at least one WMSD over the past 12 months (9). Other research literature identified the lower back (29–64%), neck (34–63%), and shoulders (17–46.7%) as the most commonly impacted body parts among nurses (11, 12, 18).

Many occupational activities and tasks, such as bending, bedside wound dressing (19), in addition to sponging, pushing beds, transferring patients, helping patients walk, lifting, and carrying, influence the prevalence of WMSDs among nurses (20). However, age, sex, work experience, work status, and being overweight were personal factors associated with WMSDs among nurses (19). Furthermore, shift work, nurse shortages, poor working conditions, and long work hours were considered organizational work factors strongly associated with WRMSD (2).

Several consequences have been associated with WMSDs, including increased absenteeism among nursing professionals, as well as temporary and permanent work limitations that can lead to career changes, work restrictions, high treatment costs, disability, and low quality of patient care (2, 21). In addition, WMSDs cause poor performance, low productivity toward patient care, temporary and permanent work limitations that lead to career changes and early retirement (19, 21). Despite the vital role of nurses in delivering patient care, the prevalence and associated factors of WMSDs among ICU and ER nurses in Palestine are poorly understood. This knowledge gap hinders the development of effective prevention strategies and interventions to safeguard the physical well-being and occupational health of nurses in these settings. This study aims to investigate the prevalence and associated factors of WMSDs among nurses working in the ICU and ER departments in Palestinian public hospitals. Additionally, the study aims to identify the body parts (patterns or anatomical areas) most affected by WMSDs and assess the consequences of WMSDs among nurses in these units.

## Methods

### Study design and settings

The study is a descriptive, multicenter cross-sectional design that was conducted among nurses working in the ICU and ER departments at four public hospitals in Palestine. The hospitals were selected to provide comprehensive data coverage across the entire West Bank, including the northern, central, and southern districts.

### Population and sample size

A total population (census) sampling strategy was employed in this study to ensure comprehensive coverage of the target population and avoid potential sampling bias associated with selective participant recruitment. Therefore, all registered nurses who met the predefined inclusion criteria and were employed in the ICU or ER departments across the four selected hospitals during the study period were invited to participate. The total eligible population consisted of 183 nurses, including 99 nurses working in ICUs and 84 nurses working in ER departments. By adopting a census strategy rather than selecting a subset of participants, the study aimed to obtain data from the entire accessible population within the selected settings, thereby strengthening the representativeness, completeness, and generalizability of the findings to the study population.

### Exclusion and inclusion criteria

The study included all registered nurses with at least 1 year of experience in ICU or ER units at the selected hospitals. Nurses who did not complete the questionnaire, pregnant nurses, those on sick leave or annual leave during the study period, and those who refused to participate were excluded from the study. Additionally, nurses suffering from rheumatic disorders, those who had encountered accidents or trauma, or who had recently undergone major musculoskeletal surgery and rehabilitation were not included.

### Data collection and measurement instrument

Data were collected from nurses working in ICUs and ER departments at the selected hospitals during the study period from 1 to 30 April 2024. Prior to distribution, the research team coordinated with head nurses and ward supervisors to facilitate access to eligible nursing staff. A paper-based self-administered questionnaire was then distributed directly to participants during their working hours.

The study utilized a self-administered questionnaire comprising five sections. The first section focused on socio-demographic data, such as gender, age, educational level, height, weight, body mass index (BMI), marital status, and years of experience. The second section included work-related factors, such as the current working department, current position (head nurse or bedside nurse), number of weekly work shifts, number of monthly night shifts, overtime work (extra jobs), the number of nurses on the same shift, break times, and dominant hand.

The third section consisted of a survey instrument adapted from the Musculoskeletal Discomfort Model, based on the Nordic Musculoskeletal Questionnaire (NMQ) (22). This scale measures the 12-month musculoskeletal pain complaints, as well as functional limitations caused by these complaints. The NMQ scale includes a body chart showing nine anatomical areas (neck, upper back, lower back, shoulders, elbows, wrists/hands, hips/thighs, knees, and ankles/feet). The questions help respondents precisely identify the presence of musculoskeletal symptoms at any of the nine anatomical areas. The nurses checked “yes” when asked if they had experienced any of the WMSD symptoms (pain, numbness, tingling, aching, stiffness, or burning) at any anatomical part during the previous 12 months, and “no” if not. The validated Arabic version was developed by Aldhabi et al. (23), and the internal consistency of the scale was excellent, with Cronbach’s alpha of 0.85. While consequences and coping strategies were evaluated in Section four of the questionnaire, including treatment-seeking, medication use, task modification, and alternative treatments. Work-related causes were assessed using the structured occupational associated factors (nurses’ work tasks) that could contribute to the development of WMSDs, such as carrying, lifting, or moving heavy materials and equipment in the department. In addition to lifting or transporting patients who require full support, bending or twisting the back awkwardly, working in the same position for extended periods, and repeatedly performing nursing tasks.

In this study, WMSDs were classified as self-reported pain, numbness, tingling, aching, stiffness, or burning in any of the nine anatomical regions measured by the Nordic Musculoskeletal Questionnaire over the previous 12 months. Regular exercise was considered structured physical activity, such as walking, running, gym training, or sports, at least 2–3 times weekly. Work experience was classified as early-career, mid-career, and experienced, corresponding to 1–4 years, 5–9 years, and 10 years or more, respectively. Completed questionnaires were checked for completeness at the time of collection to maintain data quality, and incomplete ones were rejected per the study criteria.

### Ethical considerations

This study was approved by the Research Ethics Committee at Nablus University for Vocational and Technical Education (Approval Reference: Nrs. APRIL.2023/3). The Palestinian Ministry of Health granted permission to collect data from the selected hospitals. Participation in the study was entirely voluntary, and all participants signed an informed consent form before their involvement. All data were kept strictly confidential and anonymized throughout the study.

### Data analysis

The collected data were analyzed using the Statistical Package for the Social Sciences (SPSS) Version 27. Data entry was performed and double-checked for accuracy and completeness. Descriptive and inferential statistical analyses were conducted. For descriptive statistics, frequency, percentages, mean score, and standard deviation (SD) were used to describe the study variables. For inferential analysis, chi-square tests were used to examine unadjusted associations between WMSD symptoms and sociodemographic and work-related variables. To minimize entry errors, data were entered twice by a second team member and double-checked in SPSS version 27. Statistical significance was set at *p* < 0.05.

## Results

### Socio-demographic and work-related variables of participants

Of the 183 eligible nurses working in ICU and ER departments at the four selected hospitals, 99 were ICU nurses, and 84 were ER nurses. A total of 160 nurses completed the questionnaire, including 89 ICU nurses and 71 ER nurses, yielding an overall response rate of 87.4%. Regarding participants’ characteristics, the majority were male (63.1%). Most participants were aged between 20 and 39 years, with 43.8% aged 20–29 years and 48% aged 30–39 years. More than half (55.6%) of participants were working in the ICU. Regular exercise was not common, as most nurses (76.9%) did not engage in regular physical activity. Work experience among participants was approximately evenly distributed, with roughly one-third in each category (1–4 years, 5–9 years, and ≥10 years). Furthermore, the majority of participants (65.6%) worked more than three shifts per week. The frequency of night shifts varied, with 36.3% having four to five nights per month. During shifts, nurses typically worked alongside 1–4 colleagues, with 30.6% working with three colleagues. Break durations varied considerably, with half of the nurses reporting no breaks during their working hours (Table 1).

**Table 1:** Socio-demographic and work-related variables of participants (*n* = 160).

Socio-Demographic and work-related variables		*n*	%
Gender	Male	101	63.1
	Female	59	36.9
Age	20–29 years old	70	43.8
	30–39 years old	77	48.0
	40–49 years old	10	6.3
	50–59 years old	3	1.9
Department	ICU	89	55.6
	ER	71	44.4
Educational level	Nursing diploma	21	13.1
	Bachelor’s	109	68.1
	Master’s or higher	30	18.8
BMI (Kg/m^2^)	<18.5 (underweight)	5	3.1
	18.5–24.9 (normal)	73	45.6
	25–29.9 (overweight)	59	36.9
	>30 (obese)	23	14.4
Marital status	Single	45	28.1
	Married	115	71.9
Years of experience	1–4	55	34.4
	5–9	57	35.6
	≥10	48	30.0
Engage in regular physical exercise	Yes	37	23.1
	No	123	76.9
Number of shifts per week	≤3	55	34.4
	>3	105	65.6
Number of night shifts per month	Zero	13	8.1
	2–3	28	17.5
	4–5	58	36.3
	6–7	61	38.1
Number of nurses in the same work environment shift	1	40	25.0
	2	28	17.5
	3	49	30.6
	4	43	26.9
Period of breaks during work (minutes)	Zero	82	51.2
	10	7	4.4
	15	26	16.2
	30	31	19.4
	>30	14	8.8

### WMSDs prevalence among participants

A total of 152 nurses (95.0%) reported experiencing WMSDs over the last year, with prevalence higher among ICU nurses (*n* = 86, 56.6%) compared to ER nurses (*n* = 66, 43.4%). The most commonly affected part was the lower back (85.0%), followed by the shoulders (77.0%) and the neck (71.3%), while elbows were the least affected region (27.5%). Overall, the findings indicated that the spine (lower and upper back, neck) and upper extremity joints (shoulders) were the most commonly affected body regions among participants (Table 2).

**Table 2:** Prevalence of WMSDs according to body region.

Body part (patterns)	*n*	%
Lower back	136	85.0
One or both shoulders	123	77.0
Neck	114	71.3
One or both Knees	95	59.4
Upper back	83	51.9
One or both wrists/hands	79	49.4
One or Both Ankles/Feet	74	46.3
One or Both Hips/Thighs	71	44.4
One or both elbows	44	27.5

When comparing ICU and ER departments, significant differences were observed only in knee and ankle/foot problems. ER nurses reported a higher prevalence of both knee problems (51.6% vs. 48.4%, *p* = 0.035) and ankle/foot problems (54.1% vs. 45.9%, *p* = 0.017) compared to ICU nurses, while no significant differences were found in other body regions (Table 3).

**Table 3:** Association between the affected body part and the department name.

Variable	One or Both Knees				*X* ^2^	*p*-value
	Yes		No			
	n	%	n	%		
Department name						
ICU	46	48.4%	43	66.2%	4.917	0.035*
ER	49	51.6%	22	33.8%		

Variable	One or both ankles/Feet				*X* ^2^	*p*-value
	Yes		No			
	*n*	%	*n*	%		
Department name						
ICU	34	45.9%	55	64.0%	5.225	0.017*
ER	40	54.1%	31	36.0%		

*X*^2^: Chi-square test, *Significant at *p* < 0.05, ER, emergency room; ICU, Intensive care unit.

### Consequences and coping strategies of WMSDs among nurses

**Figure 1:**
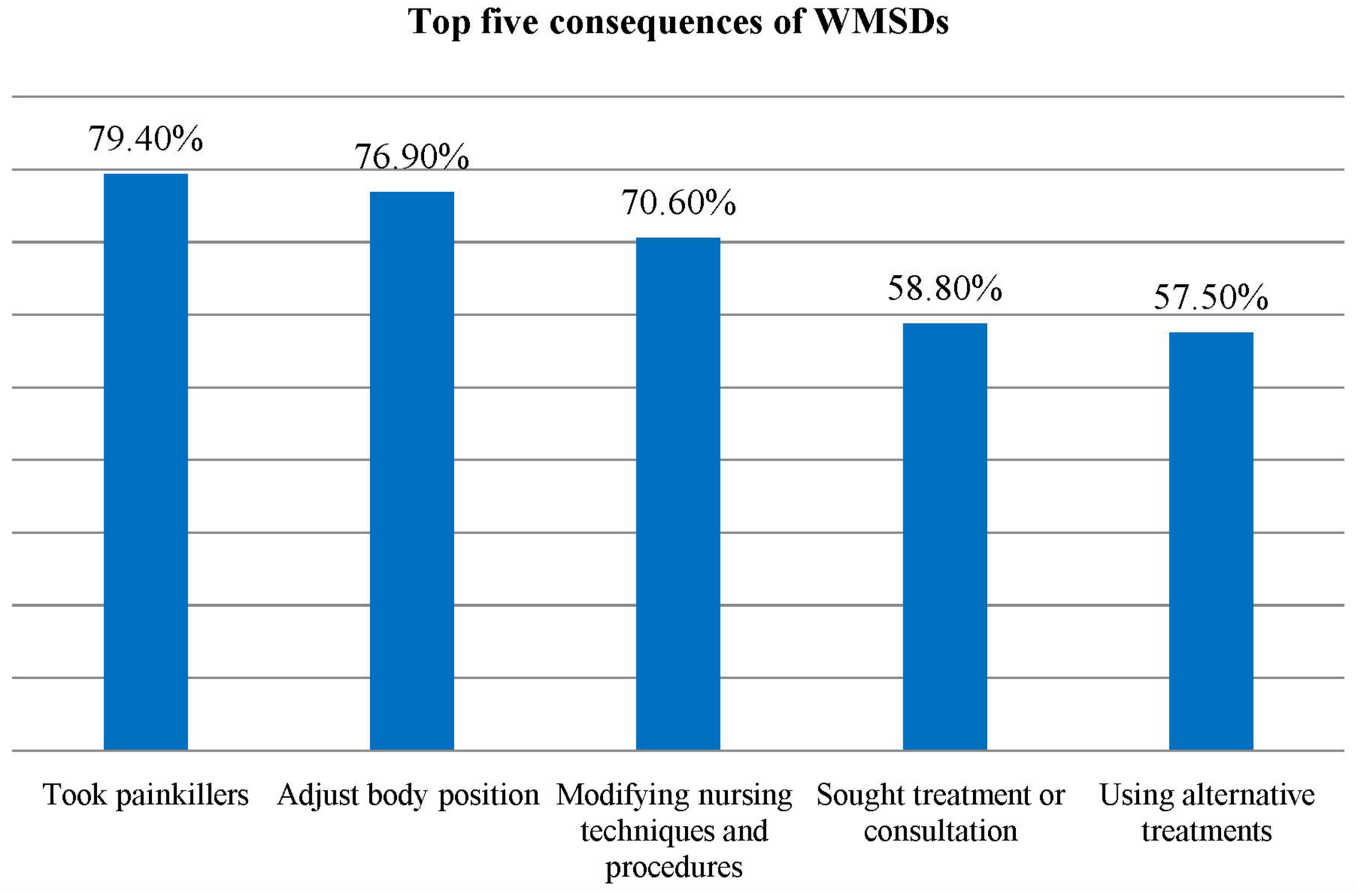
Top five consequences of WMSDs.

This study identified the most common consequences and coping strategies taken by affected nurses to adapt and overcome these symptoms. The majority of affected nurses (*n* = 130, 81.3%) reported that WMSDs prevented them from performing normal work tasks in the past 12 months. In response, the most common coping strategies were taking prescribed medication or painkillers (*n* = 127, 79.4%), adjusting body positions to avoid re-injury (*n* = 123, 76.9%), and modifying nursing techniques to distribute workload more effectively (*n* = 113, 70.6%). Fewer nurses sought formal treatment and consultation (*n* = 94, 58.8%) and used alternative therapies such as massage, herbal remedies, or physical therapy (*n* = 92, 57.5%) (Figure 1).

### Causes and work-related factors of WMSDs

The most frequently reported work-related causes of WMSDs included repeatedly performing nursing tasks (94.4%), continuing to work despite pain or injury (93.1%), and inadequate rest or breaks during shifts (91.9%). Physical and patient-related demands were also commonly reported, including working at physical limits (89.4%), high patient loads (88.8%), prolonged static postures (85%), and lifting or transporting patients who require full support (manual patient handling) (83.1%). Conversely, the least reported factors were not receiving help when handling heavy patients (62.5%) and carrying or moving heavy materials and equipment (58.1%) (Table 4).

**Table 4:** Self-reported activities or job associated factors leading to WMSDs.

Self-reported job associated factors or activities leading to WMSDs	*n*	%
1. Repeatedly performing nursing tasks	151	94.4
2. Continue to work despite injury or harm	149	93.1
3. Not having enough rest/breaks during the day	147	91.9
4. Working at or near physical limits	143	89.4
5. Treating a large number of patients daily	142	88.8
6. Working in the same position for long periods	136	85.0
7. Inadequate training in injury prevention	134	83.8
8. Bending or twisting back in an awkward way	134	83.8
9. Lifting or transporting patients who require full support	133	83.1
10. Working with confused or agitated patients	131	81.9
11. Perform manual nursing techniques	130	81.3
12. Assist patients during walking or transferring to or from bed	126	78.8
13. Lack of assistive devices and equipment (wheelchair, walking walker)	125	78.1
14. Sudden unexpected movements or falls by patients	119	74.4
15. Over time, irregular shift, length of workday	116	72.5
16. Straining the body to try to reach distant equipment or tasks	116	72.5
17. Do not change the height of the base or bed while working with patients	109	68.1
18. Working in uncomfortable or cramped positions (cramped spaces).	109	68.1
19. Not getting help while dealing with a heavy patient	100	62.5
20. Carrying, lifting, or moving heavy materials and equipment.	93	58.1

### Associated factors associated with WMSD symptoms

For inferential analysis, chi-square tests were used to examine unadjusted associations between WMSD symptoms and sociodemographic and work-related variables. Given the very high prevalence of WMSDs and the small number of WMSD-negative participants, adjusted logistic regression was not considered statistically stable. Therefore, the findings should be interpreted as unadjusted associations rather than independent causal risk factors. Data analysis identified regular exercise and years of experience as factors significantly associated with WMSDs in the last 12 months. Among nurses with WMSDs, only 21.1% exercised regularly compared to 78.9% who did not exercise (*p* = 0.017). Regarding work experience, nurses with 5–9 years reported the highest significant prevalence of WMSD symptoms (37.5%), with none in this group being symptom-free, compared to those with 1–4 years (32.2%) and ≥10 years (30.3%) of experience (*p* = 0.029). The within-department prevalence of pain, discomfort, and numbness due to work was 96.6% among ICU nurses, 86 of 89, and 93.0% among ER nurses, 66 of 71. The overall difference between departments was not statistically significant, chi-square = 1.121, *p* = 0.468 (Table 5).

**Table 5:** Factors associated with WMSDs in the past 12 months among participants’ socio-demographic and workplace-related variables.

Variables	The prevalence of pain, discomfort, and numbness due to work				*X^2^*	*p*-value
	Yes		No			
	*n*	%	*n*	%		
Gender						
Male	94	61.8%	7	87.5%	2.149	0.260
Female	58	38.2%	1	12.5%		
Age group						
20–29 years old	64	42.1%	6	75.0%	3.500	0.321
30–39 years old	75	49.3%	2	25.0%		
40–49 years old	10	6.6%	0	0.0%		
50–59 years old	3	2.0%	0	0.0%		
Department						
ICU	86	56.6%	3	37.5%	1.121	0.468
ER	66	43.4%	5	62.5%		
Work experience						
1–4 years	49	32.2%	6	75.0%	7.113	0.029*
5–9 years	57	37.5%	0	0.0%		
≥10 years	46	30.3%	2	25.0%		
Number of shifts per week						
≤3	52	34.2%	3	37.5%	0.036	0.560
>3	100	65.8%	5	62.5%		
Current position						
Head of Department	9	5.9%	0	0.0%	0.502	0.623
Registered Nurse	143	94.1%	8	100.0%		
Exercise regularly						
Yes	32	21.1%	5	62.5%	7.344	0.017*
No	120	78.9%	3	37.5%		
BMI						
Underweight	5	3.3%	0	0.0%	2.568	0.463
Normal	71	46.7%	2	25.0%		
Overweight	54	35.5%	5	62.5%		
Obesity	22	14.5%	1	12.5%		

*Significant at *p* < 0.05, BMI, body mass index; ICU, intensive care unit; ER, emergency room; *X*^2^, Chi-square test.

## Discussion

The findings of this study revealed a concerning prevalence of WMSDs among nurses working in ICU and ER departments, with 95% of participants reporting WMSD symptoms in the past 12 months. This rate was notably higher than prevalence rates reported in other Arab countries, including Saudi Arabia (63.8%) (12), Tunisia (48.1%) (24), and Morocco (84%) (25). Also, our result was higher in the prevalence of WMSDs among nurses in Egypt, which ranged between 62.3% (7), 88% (9), and 92.3% (10), while it was 71.3% in Lebanon (26). These studies generally focus on hospital nurses broadly, whereas the current study specifically targeted two of the most demanding departments (ICU and ER), which may explain the higher prevalence observed. Some studies identified ER nurses as being the most affected compared to nurses in other wards (9, 15, 17). For instance, another study reported the highest prevalence of musculoskeletal symptoms among ER nurses in Egypt (9), and the study in Taiwan found that ER nurses faced a significantly elevated WMSDs compared to ICU nurses (15). However, none of these studies were conducted in the Arab region, directly comparing ER and ICU nurses in terms of WMSDs. In contrast, our study found that WMSDs were more common among ICU and ER nurses (56.6 and 43.4%, respectively).

The present study found that the lower back was the most affected area by WMSDs, which is aligned with previous worldwide studies (4, 19, 27), and in Arab countries (7–10, 28). Excessive workload, unfavorable work environment, working in static or poor back postures for a long period, and repetitive tasks may be contributing factors (19). Additionally, activities such as assisting patients with repositioning in bed, moving them out of bed, and lifting them from the floor were frequently identified as contributors to back pain among nurses (29). Another possible explanation is that reduced lumbar multifidus muscle quality may compromise spinal stabilization, increasing the risk of low back pain among healthcare professionals, including nurses (30). In the present study, over 80% of nurses reported bending or twisting their backs awkwardly, in addition to lifting and transporting patients requiring full support, both of which are well-recognized associated factors for low back WMSDs.

Beyond the lower back, the shoulders (77.0%) and neck (71.3%) were the second and third most affected body regions in the present study. This pattern is consistent with global evidence from a recent worldwide meta-analysis of 90 studies, which identified lower back, neck, and shoulder as the three most commonly affected body areas among nurses globally (3). A high prevalence of neck and shoulder WMSDs among nurses has also been previously reported in the Arab region, where Jordanian nurses reported neck issues as the most common upper quadrant WMSDs, followed by upper back and shoulder disorders (11). Similarly, neck and shoulders were the second and third most affected regions among nurses in Saudi Arabia (31), while in Egypt, some studies (9, 32) identified neck as the second most affected region and the knee as the third. Notably, a recent study among nurses in conflict-affected hospitals in Gaza (14) identified neck (69.2%), lower back (68.0%), and shoulders (64.5%) as the three most commonly affected body regions, further reinforcing the vulnerability of these body regions among Palestinian nurses regardless of setting. These results may be linked to extended periods of static posture, repetitive motions of the upper limbs, and the physical demands associated with patient care tasks, such as repositioning and monitoring individuals in ICU and ER environments.

The present study identified the most common coping strategies used by nurses to manage pain or injuries related to WMSDs. Reactive coping strategies were predominant, with more than three-quarters of participants regularly using prescribed medications and painkillers to relieve pain, while a similar proportion adjusted their body positions or modified nursing techniques to distribute workload more effectively. Preventive strategies were less commonly reported, including seeking assistance with heavy patient handling, repositioning patients, and receiving formal training on injury prevention. The reliance on reactive rather than preventive coping strategies is concerning, as prior literature has highlighted regular physical exercise as an effective preventive strategy for reducing WMSD associated factors (19). Given that 76.9% of nurses in the present study did not exercise regularly, there is a clear need to promote preventive strategies, including physical activity, within this population.

Although WMSDs were highly prevalent among both ICU and ER nurses, the overall difference in WMSD prevalence between departments was not statistically significant. However, the specific anatomical regions that differed significantly, particularly knee and ankle or foot symptoms, were more frequently reported among ER nurses. Similarly, ankle/foot issues have been reported to be significantly more common among nurses in the emergency department than in other nursing departments in Egypt (9). This difference may be attributed to the fact that ER nurses generally face a more dynamic, unpredictable, and physically demanding nature of ER work compared to ICU settings. The lower limbs, particularly the knees and ankles/feet, are subjected to increased biomechanical stress in the ER, where nurses frequently stand for extended periods, move swiftly between patients, and perform repetitive actions such as pushing, pulling, bending, and lifting during emergencies (33). It was previously reported that tasks such as repetitive movements, lack of lifting equipment, pushing/pulling, and sustained standing were associated with WMSDs at the knees and ankles/feet (28). Conversely, ICU nurses typically work in a more stable and less mobile environment, caring for mostly bedridden patients requiring close observation, although physically demanding tasks remain common in this setting (34).

The current study found a significant association between WMSD symptoms and both exercise habits and work experience. Interestingly, nurses with 5–9 years of experience reported the highest WMSD symptoms compared to both less experienced (1–4 years) and more experienced (≥10 years) colleagues, with none in the 5–9-year group being symptom-free. This suggests that mid-career nurses may represent a particularly vulnerable group. While early-career nurses may be more physically resilient with less cumulative exposure, more experienced nurses may have developed better body mechanics and adaptive strategies over time. In contrast, mid-career nurses may have accumulated sufficient occupational strain without yet having fully developed these protective habits, combined with increased workload and responsibilities compared to junior staff. Our findings are consistent with other previous studies that showed years of experience were previously identified as an associated factor for WMSDs among Lebanese nurses with 5–10 years of experience compared to those with less than 5 years (26), and among Iranian nurses, where WMSDs increased significantly with increasing work experience (27). However, Egyptian nurses with more than 10 years of experience were reported to be at higher risk (9), suggesting that the relationship between experience and WMSDs may vary across different healthcare contexts and warrants further investigation.

The present study also identified regular exercise as a significant protective factor against WMSDs, with nurses who did not exercise regularly being at a higher associated factor of developing symptoms. Notably, most Palestinian nurses in the present study reported not engaging in regular physical activity, which is particularly concerning given the physically demanding nature of ICU and ER work. Regular physical exercise is believed to strengthen musculoskeletal structures, improve flexibility, and reduce cumulative physical fatigue, thereby lowering the factor of occupational injuries (35). These findings are consistent with previous studies highlighting that nurses who do not engage in regular physical exercise are more likely to experience WMSDs (19, 36).

### Limitations of the study

This study has several limitations that should be considered when interpreting the findings. First, data collection relied on self-administered questionnaires, which may introduce recall bias in participants’ responses. Second, psychosocial factors such as job stress, workload perception, and social support as well as behavioral factors such as smoking and alcohol use, were not collected in the original questionnaire, which may have provided a more comprehensive understanding of WMSDs among nurses. Third, this study was limited to governmental hospitals, which may affect the generalizability of the findings to private hospitals, southern regions of the West Bank, or conflict-affected areas such as Gaza, where occupational conditions may differ substantially. Future studies should therefore include multi-region and multi-sector samples. Finally, the cross-sectional design prevents the establishment of causal relationships between the examined variables. This limitation could be addressed in future research through longitudinal or prospective cohort designs.

## Conclusion

This study demonstrated a very high prevalence of WMSDs among Palestinian ICU and ER nurses, affecting 96.6% of ICU nurses and 93.0% of ER nurses. The overall prevalence did not differ significantly between departments. The lower back, shoulders, and neck were the most commonly affected anatomical regions. ER nurses reported significantly higher knee and ankle or foot symptoms, while regular exercise and work experience were associated with WMSD symptoms in unadjusted analyses. These findings highlight the need for preventive ergonomic interventions, adequate staffing, regular breaks, and workplace-based musculoskeletal health programs for nurses in high-demand clinical settings. Policymakers should prioritize the development of occupational health policies that emphasize safe staffing levels and preventive healthcare for nurses, while occupational safety education should be incorporated into both undergraduate curricula and continuing professional development. Further longitudinal research is needed to establish causal relationships and evaluate the effectiveness of preventive interventions in this population.

## Data Availability

The raw data supporting the conclusions of this article will be made available by the authors, without undue reservation.
